# Insecticide regulations need to consider impacts on conservation adoption

**DOI:** 10.1093/biosci/biag062

**Published:** 2026-07-02

**Authors:** Tristan A Barley, Lisa A Schulte, Amy L Toth, Matthew O’Neal, Adam G Dolezal

**Affiliations:** Department of Entomology, University of Illinois Urbana-Champaign, 505 S. Goodwin Avenue, 320 Morrill Hall, M/C 118 Urbana, IL 61801,USA; Natural Resource Ecology and Management and Bioeconomy Institute, Iowa State University, 617 Bissel Road., 1140 Biorenewables Research Lab, Ames, IA 50011-1098, USA; Ecology, Evolution, and Organismal Biology, Iowa State University, 251 Bessey Hall, 2200 Osborn Dr #251, Ames, IA 50011, USA; Plant Pathology, Entomology, and Microbiology, Iowa State University, Advanced Teaching and Research Building, 2213 Pammel Drive, Ames, IA 50011, USA; Plant Pathology, Entomology, and Microbiology, Iowa State University, Advanced Teaching and Research Building, 2213 Pammel Drive, Ames, IA 50011, USA; Department of Entomology, University of Illinois Urbana-Champaign, 505 S. Goodwin Avenue, 320 Morrill Hall, M/C 118 Urbana, IL 61801,USA

**Keywords:** policy, conservation, insecticides

## Abstract

The US Environmental Protection Agency implemented a new insecticide strategy requiring site-specific changes to their use. An updated strategy was urgently needed, given the scientific evidence linking pesticides with precipitous declines in pollinators and increasing numbers of insects added to or proposed for listing under the Endangered Species Act. Protecting nonpest insects requires all hands on deck; however, the new strategy threatens to undermine support from a key set of hands: farmers who use pesticides, and who are crucial partners for achieving conservation goals. Care should be taken to align the insecticide strategy with the USDA Conservation Reserve Program (CRP), a popular program that provides crucial financial support for farmland owners to voluntarily engage in conservation, including insect conservation. We spotlight the CRP Prairie Strips (CP-43) practice to exemplify problems with the new insecticide strategy and recommendations to improve the policy for both farmers and the environment.

In May 2025, the US Environmental Protection Agency (EPA) released an updated insecticide strategy (USEPA 2025a) to reconcile their policy on registering and evaluating insecticides per requirements of the Endangered Species Act (ESA). This policy integrates information from multiple US federal agencies, including the EPA, which regulates pesticide use; the US Fish and Wildlife Service (FWS), which designates critical habitat for endangered species; and the US Department of Agriculture (USDA), which supports voluntary conservation action by farmers and farmland owners. An updated strategy was urgently needed, given the well-documented, scientific evidence linking pesticides with precipitous declines in pollinators and other beneficial insects (Brittain et al. 2010, Janousek et al. 2023, Edwards et al. 2025). This new iteration attempts to more concretely identify mitigations to protect numerous ESA-listed species (USEPA 2025a.). Additionally, this new regulatory document creates a scorecard system to allow farmers to combine conservation practices to remain in compliance. The wording of EPA’s new strategy, however, is unclear regarding compliance with the ESA. This creates uncertainty as to whether additional mitigation efforts, at the expense of farmers, may be required if future ESA listings mandate extra protections.

Vagueness in the EPA’s policy may deter a crucial target group (e.g., farmers who use pesticides) from participating in voluntary conservation initiatives. Research on the human dimension of conservation indicates that farmers and farmland owners are less likely to participate in government conservation programs that provide a low return on investment, that frequently change, or that are overly burdensome (Atwell et al. 2010). Uncertainty tends to dissuade farmers from practicing conservation, especially when the farmers are growing low-value crops that require careful financial management to achieve profitability. It is unclear whether future ESA listings and critical habitat designations will mandate the establishment of additional buffers around existing conservation practices adjacent to agricultural fields (figure 1). Depending on how a buffer is defined, it could require removing land from production or limiting management practices on adjacent cropland. These changes would be a burden to farmers, dissuading them from participating in voluntary conservation. We propose modifying the strategy to clarify the terminology and create certainty regarding the protective status for voluntary conservation programs, such as the USDA Conservation Reserve Program (CRP). Currently, there is no language in the EPA's documents that specifies that conservation programs could not be listed as critical habitat in the future, which could require extra mitigations. Even if there is no precedent for such protection and an internal understanding within the EPA and the FWS that conservation practices would not be labeled as critical habitat, a lack of documentation does not dispel a perception within the farming communities that the CRP and other practices could be listed for protection. Germane to this point, the EPA acknowledges that the practices and regulations outlined in this document are dynamic and are undergoing additional reviews.

**Figure 1:**
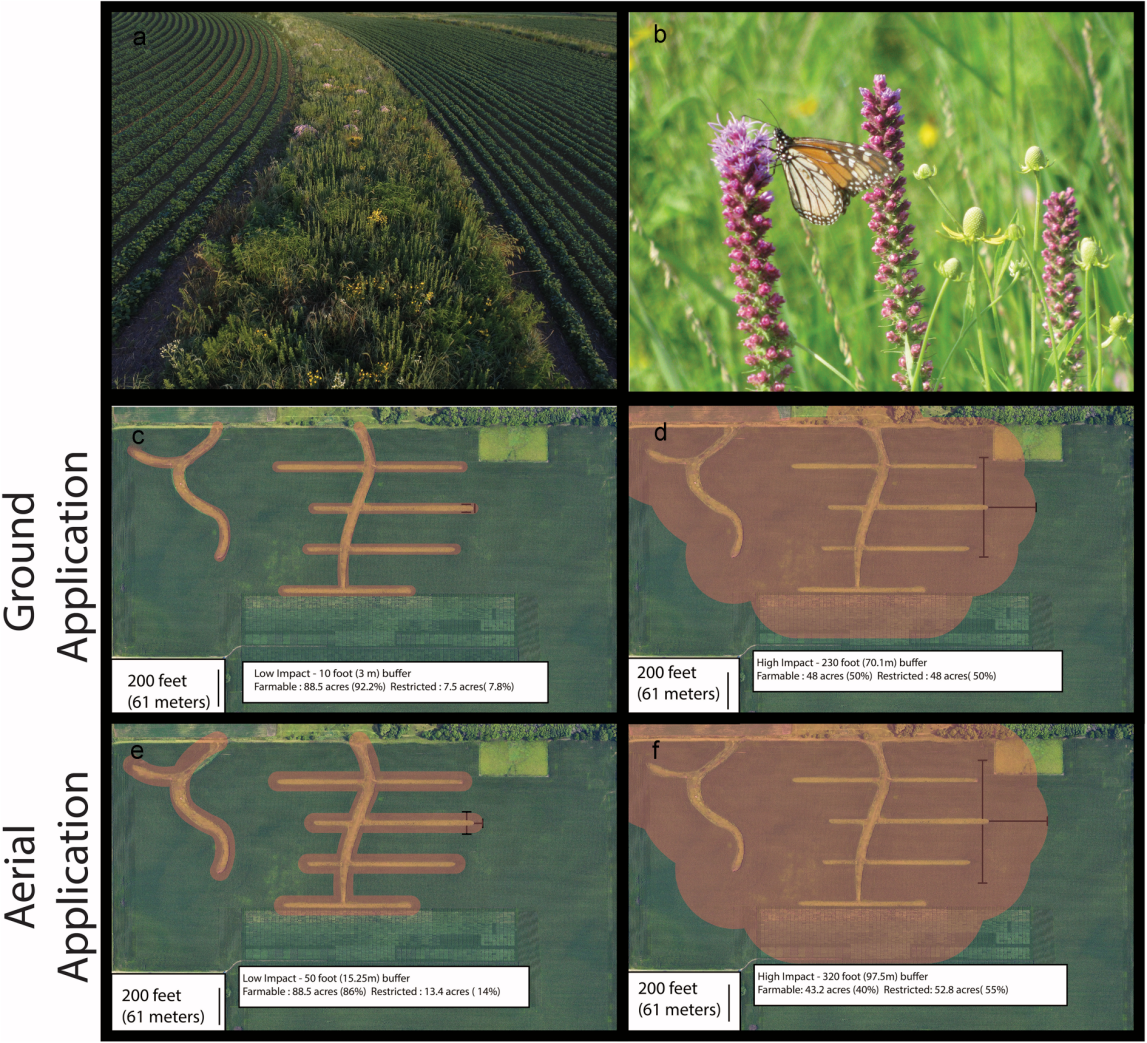
A model cropfield with CRP Prairie Strips (CP 43) and Grassed Waterways (CPS 412), with buffer sizes determined using EPA recommendations (table 4.1 in USEPA 2024.). Mitigation strategies can reduce the percentage of land required for a buffer; this example represents a worst-case scenario, in which the farmer has no mitigations. (a)–(b) Example prairie strip running through crops; native floral species attract a multitude of pollinators. (c)–(d) Buffers (dark brown) required for ground applications around example prairie strips and grassed waterways (light brown), assuming a low (c) or high (d) impact insecticide. (e)–(f) Buffers required for aerial applications around prairie strips, assuming a low-impact (c) or high-impact (d) insecticide.

The scientific community has an opportunity to engage with the EPA through public comment periods and professional societies to make these concerns known so that the insecticide strategy can meet the needs of pollinators, farmers, and regulators. We expect that these changes will facilitate alignment among crop production and insect conservation goals on privately owned land and galvanize the all-hands-on-deck approach advocated for the conservation of the monarch butterfly on both public and private land (Thogmartin et al. 2017).

Beyond insects designated as threatened or endangered by the FWS, pollinators, in general, are declining across the United States because of multiple often interrelated stressors, including parasites, pathogens, disruptions due to climate change, pesticide use, and habitat loss (Wagner et al. 2021, Edwards et al. 2025). These stressors often coincide on farms (Wagner et al. 2021). Although the effects of these stressors are complex and context dependent, restoration and reconstruction of quality habitat is crucially important to combat declines (Morandin et al. 2014, Edwards et al. 2025). The FWS has the role of defining critical habitat for threatened and endangered species and requires protection of those habitats to promote conservation of imperiled taxa. Critical habitats have already been described for some species listed under the ESA; however, even these designations can be ambiguous. For example, critical habitat proposed for *Bombus affinis* (the rusty patched bumble bee) in November 2024 lists 1,635,746 acres across 14 units (locations) ranging from Minnesota to West Virginia ([FWS 2024] Federal Register 2024—27316 [89 FR 93245]). These designations are broad and provide exemptions to buildings and other constructions but not other surrounding land types or for CRP practices. Notably, the Natural Resources Conservation Service's Wetlands Reserve Program (NRCS 2025) land is not exempt ([FWS 2024] Federal Register 2024–27316 [89 FR 93,245]). If these habitats are not given an exemption, then how likely is the CRP to receive leniency? Without an explicit exemption, it is unclear whether land enrolled in the CRP (either previously existing or through future leases) would classify as critical habitat and, therefore, require the landowner to provide additional protections. The critical habitat proposal states ([FWS 2024] Federal Register 2024—27316 [89 FR 93245]) that special management considerations may allow for the use of pesticides in adjacent crops while also noting that critical habitat areas were selected away from large-scale agriculture. These ambiguities and contradictory sections only add to the confusion. Without explicit clarification, farm landowners enrolled in the CRP and other conservation practices may be required to provide additional mitigation efforts at their own expense.

As an illustrative example of the issues that could arise, consider *B. affinis*'s critical habitat Unit 13, located in Johnson County, Iowa. As of 2020, Iowa was one of the states with the highest CRP adoption, with an average of approximately 17,000 acres enrolled in various CRP programs per county, approximately 2,200 of which consisted of CP 42 (pollinator habitat), habitat explicitly created for pollinators (FSA 2025). Johnson County had approximately 10,408 acres of CRP enrollment in 2020 and falls within the top 7% of counties for enrollment nationwide (FSA 2025). A majority (approximately 54%) of land use in the county is dedicated to agriculture, including land that falls within the Unit 13 critical habitat designation (USDA-NASS 2024). Because Iowa is at the forefront of agriculture intersecting pollinator habitat and because important biological features for *B. affinis*, such as overwintering, nesting, and foraging habitat, may be found throughout this unit, the critical habitat designation in this area could have consequences for regulatory interpretations across the state ([FWS 2024] Federal Register 2024—27316 [89 FR 93245]). Furthermore, special management considerations or protections may be required in this unit to alleviate the impacts from stressors, including pesticide applications ([FWS 2024] Federal Register 2024—27316 [89 FR 93245]). In the future, farmers considering adoption of CP 42 and 43 (prairie strips), or both may wonder whether this CRP land falls within critical habitat designations. Would a farmer in this scenario be required to create additional buffers to protect a newly enrolled CRP land from pesticide exposure if it falls within the critical habitat for *B. affinis*? The EPA’s insecticide strategy states that CRP land can be a part of a buffer from pesticide drift (USEPA 2025a), but the critical habitat designation provided by the FWS does not state that the CRP is exempt and, instead, notes that management should be considered in these areas. It further notes that the Wetlands Reserve Program is not exempt, leaving it unclear whether CRP enrollment is similarly not exempt. If a farmer were to interpret these regulations as requiring extra protections for CRP land, this could affect how they apply insecticides and could potentially cause existing mitigations to no longer be applicable (figure 2). This ambiguity may disincentivize farmers from newly enrolling or extending leases for CRP practices.

**Figure 2:**
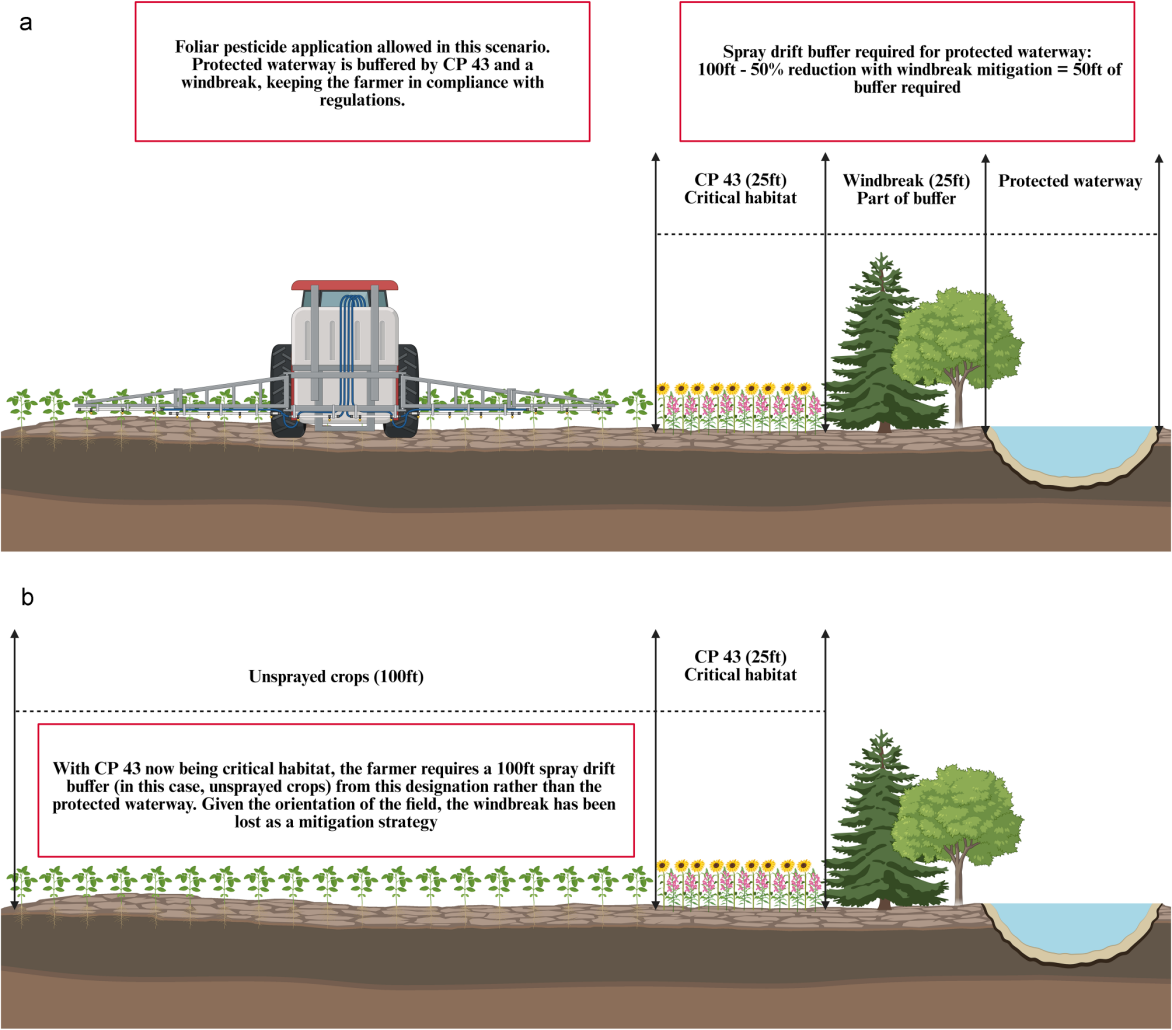
An example of how critical habitat designations could alter buffer sizes and mitigations, based on figures 6 and 7 in the insecticide strategy (USEPA 2025a.), created in BioRender (https://BioRender.com). (a) A protected waterway necessitates a 100-foot spray drift buffer. The farmer has a riparian buffer that functions as a windbreak, which reduces the needed buffer by 50% (i.e., 50 feet of buffer is now required; see table 10 in USEPA 2025a). In this example, the 25 feet of CP 43 and the 25 feet of riparian buffer can act as the required 50 feet of spray drift buffer (table 11 in USEPA 2025a.), and the farmer can apply insecticides to the entirety of their field. (b) This example posits that CP 43 has been designated as critical habitat. Given the orientation of the field, the riparian buffer does not provide mitigation benefits (i.e., a reduction in the size of the buffer), and the farmer will be required to not spray 100 feet of crops adjacent to CP 43 to be in compliance.

As is illustrated above, our primary concern is that this ambiguity found in the EPA’s new insecticide strategy will make it difficult for landowners to determine the protection status of voluntary conservation practices. A joint statement by the EPA and the FWS attempted to address these concerns, noting that the EPA is compliant in its insecticide strategy with FWS regulations and risk assessments (USEPA 2025a. pp. 6, 15; USEPA 2025b.). Furthermore, the insecticide strategy notes that noncrop habitat (e.g., areas planted with pollinator-friendly flora rather than crops) may attract endangered or threatened insect species, whose presence is believed to be more beneficial than the risk of their potential exposure to insecticides from nearby cropland. This exposure is already addressed by the EPA, because insecticide applicators are required to apply active ingredients such that drift does not occur. However, it is unclear whether the EPA will demand even more of farmers to prevent insecticides from encountering endangered wildlife. If the strategy requires farmers to limit pesticide use within cropland adjacent to an edge of field practice, the burden of conservation will extend beyond land taken out of production.

Although we laud efforts by the EPA and the FWS to apply the best available scientific information, the strategy is confusing to navigate, and its wording is unclear. The insecticide strategy states that changes to critical habitat designations could alter mitigation strategies and depends on future consultations with the FWS. Although participation in some conservation programs may meet all mitigation requirements for the use of a particular insecticide, reviews of these programs are ongoing. This ambiguous language and references to analyses and supporting documents have yet to be released, creating uncertainty regarding whether any insecticide use is in compliance.

Creating habitat adjacent to agricultural fields is a common approach for conserving insects and is becoming more popular in the US Midwest. Prairie strips, discussed in more detail in Toth and colleagues (2026), provide an example of how the insecticide strategy could be at odds with achieving insect conservation goals. Prairie strips are linear plantings of perennial, herbaceous, native plant species located within and at edges of annual crop fields (usually corn or soybean monocultures) to provide multiple benefits. This practice (CRP practice CP 43) addresses farmer concerns for soil and water conservation (Schulte et al. 2017) while increasing the abundance and diversity of beneficial insects, including predators of crop pests (Cox et al. 2014), wild bees (Kordbacheh et al. 2020), and monarch butterflies (Stephenson et al. 2025). Prairie strips also improve the health and productivity of honey bees (Zhang et al. 2023). On farms without the flowering plants common in prairies, honey bees experience a late summer famine (Dolezal et al. 2019). Pollinators in these habitats may be exposed to a wide variety of potentially harmful pesticides (Main et al. 2020); these effects are only seen up to 25 meters (82 feet) outside of agricultural fields (Goebel et al. 2022). The practice of using neonicotinoid treated seeds has not resulted in lethal levels of these insecticides being found in forb plant tissue (Hall et al. 2022). Although honey bee exposure to field-realistic concentrations of commonly used pesticides (lambda-cyhalothrin, thiamethoxam, and chlorpyrifos) in lab experiments can produce some negative interaction with other stressors (Hsieh and Dolezal 2024), field evidence broadly suggests that flowering strips next to crop fields, whether those fields contain field or fruit crops, do not increase pollinator exposure to foliar applications of pesticides (Hall et al. 2022, Graham et al. 2024, Toth et al. 2026). Overall, these practices increase the abundance, diversity, and health of pollinators, even when adjacent crop fields are managed conventionally. Similar conservation benefits have been found with other practices, such as CP 42 (Quinlan et al. 2023) and hedgerows adjacent to cropland (Morandin et al. 2014). However, there is no language in the insecticide strategy stating such an approach is exempt from being designated as critical habitat in the future. Designating these practices as critical habitat would be a burden to farmers and likely reduce participation in CP 43 and other conservation initiatives if restrictions to pesticide exposure are required (figure 1).

The tensions and trade-offs between production and conservation apply broadly to US agriculture, although the intensity varies by region. Although adoption of some conservation practices is increasing (e.g., CP 43 prairie strips), overall enrollment in the CRP peaked in 2007. Since then, millions of acres of grassland previously enrolled in the CRP have returned to cropland, resulting in substantial loss of habitat for pollinators (Otto et al. 2018). Publicly available data from the USDA indicate that 27 states contain less than 10% federal-, state-, or public-owned land, and in several highly agricultural states (e.g., Iowa, Illinois, Indiana, Kansas, and Nebraska), these spaces make up less than 3% of the total state (USDA 2025). Therefore, making appreciable strides in insect conservation will require substantial buy-in from private landowners to voluntarily engage with conservation practices such as the CRP.

Private landowners, often farmers, are crucial conservation partners who struggle to implement needed practices because of the challenging economics of farming (Atwell et al. 2010). Uncertainty regarding habitat protection requirements could dissuade private landowners from engaging with voluntary conservation practices because of concerns for added protections and burdensome changes to pesticide application requirements in the future. We expect decisions made that prioritize production and certainty will eclipse conservation if CRP practices, such as CP 43, are burdened by confusing requirements (figure 1).

Discouraging participation in voluntary conservation practices is antithetical to the intentions of the new insecticide strategy. The EPA noted that, although insecticide exposure can be problematic, habitat creation is more important in pollinator conservation efforts (USEPA 2025a), which aligns with current research (see Toth et al. 2026). The EPA further indicates that a continued review of data and updates to supporting documents is ongoing, hopefully to clarify and improve the pairing of conservation and pesticide mitigation. Although future changes present an opportunity to build upon the insecticide strategy and address concerns, reduced staffing at federal agencies is likely to negatively affect implementation of complex and shifting strategies. Many environmental policies require interagency coordination to ensure effective, lawful implementation so that the needs of people and the environment are adequately met.

Overall, insect conservation urgently requires all hands on deck so that more, much needed habitat can be returned to our landscape. In its current form, we predict that the misalignment between production agriculture and conservation will result in a substantial reduction in the amount of habitat for pollinators and other beneficial insects on farms. The best available scientific information indicates that on-farm habitat, as supported through the CRP, does not require adjusting farming practices within the adjacent cropland. Therefore, we recommend that the EPA reduce regulatory burdens on the establishment of pollinator and beneficial insect habitat in and around crop fields. We instead recommend the following: Clearly define the purpose of a buffer as a mitigation strategy, particularly in instances where a buffer could be composed of noncrop plant species that provide valuable resources to species listed under the ESA. Clearly communicate that occupancy of noncrop buffers by a threatened or endangered species would not result in practices such as CP 43 being considered critical habitat under the ESA, necessitating additional mitigations. Plain, clear language is needed to assure landowners that noncrop habitat adjacent to crop land in which insecticides are applied would not run afoul of EPA regulations. Forgo additional burdens to voluntary conservation in agricultural settings. These changes would encourage continued participation by agricultural communities in voluntary conservation practices, which is vital to meet conservation goals. The technical jargon outlined in the insecticide strategy, although it is necessary, is challenging to navigate. The EPA should work closely with extension professionals nationwide to create fact sheets for farmers, simultaneously connecting them more directly with extension offices, facilitating discourse between the farmer and the regulator. In addition to these suggestions, agencies such as the EPA often use data from these public comment periods and interactions with science policy advocates to justify changes to congressional members. Expressing these concerns to congressional offices is another avenue that can facilitate communication in these political and regulatory spaces. EPA officials should revise the insecticide strategy documents with aid of farmers and extension professionals so that the text does not inadvertently dissuade the adoption of necessary conservation practices.
